# Ultra-widefield optical coherence tomography angiography plus colour fundus photography in von Hippel‒Lindau disease: detection, classification, and correlations with findings on fluorescein angiography

**DOI:** 10.1186/s40662-026-00489-x

**Published:** 2026-05-14

**Authors:** Xiaonan Zhuang, Yao Zhou, Fengjuan Gao, Yi Xuan, Min Wang, Qing Chang, Xin Huang, Gezhi Xu, Wei Liu

**Affiliations:** 1 Eye Institute and Department of Ophthalmology, Eye and ENT Hospital of Fudan University, 83 Fenyang Road, Shanghai, 200031 China; 2 Shanghai Key Laboratory of Visual Impairment and Restoration, Shanghai, China; 3 NHC Key Laboratory of Myopia and Related Eye Diseases, Chinese Academy of Medical Sciences, Shanghai, China

**Keywords:** Retinal capillary haemangioma, Von Hippel‒Lindau disease, Optical coherence tomography angiography

## Abstract

**Background:**

To compare the utility of single-capture ultra-widefield optical coherence tomography angiography (UWF-OCTA) and UWF-OCTA plus UWF colour fundus photography (UWF-CFP) versus UWF fluorescein angiography (UWF-FA) in detecting retinal capillary haemangiomas (RCHs) in von Hippel‒Lindau disease (VHL) and to explore the associations of RCH multimodal imaging features.

**Methods:**

In this observational cross-sectional study, all enrolled eyes underwent single-capture UWF-OCTA (29 × 24 mm). RCHs suspected on UWF-CFP with eye-steering were further checked using regional UWF-OCTA scans. UWF-FA was used for comparison. Independent observers performed the RCH detection and characterisation using different imaging methods. The detection performance was compared, and logistic regression was used to identify the factors associated with leakage.

**Results:**

Thirty-nine eyes of 21 patients with VHL were included in this study. At the eye level, UWF-OCTA plus UWF-CFP exhibited a similar performance to UWF-FA in detecting RCH involvement (87.2% vs. 89.7%, *P* = 0.319) and the median number of RCHs per eye (2 vs. 2, *P* = 0.252). However, the RCH involvement rate (61.5% vs. 89.7%, *P* < 0.001) and number of RCHs per eye (1 vs. 2, *P* = 0.003) were lower with single-capture UWF-OCTA than with UWF-FA. At the RCH level, UWF-OCTA plus UWF-CFP showed slightly lower detection rates than did UWF-FA, albeit without statistical significance (86.8% vs. 93.4%, *P* = 0.057). Single-capture UWF-OCTA detected significantly fewer RCHs than did UWF-FA (51.7% vs. 93.4%, *P* < 0.001). RCHs were classified according to OCTA B-scan characteristics. Types 1 (48.8%) and 2 (18.1%) RCHs exhibited a nodular appearance with protrusion into the vitreous cavity and compression of the outer retina, respectively. Type 3 RCHs (28.3%) displayed flat growth patterns, whereas type 4 RCHs (4.7%) breached the inner limiting membrane. Logistic regression revealed that RCH size > 0.5 mm was associated with hyperfluorescence with leakage (odds ratio [OR]: 10.987; 95% confidence interval [CI]: 1.747 to 69.090; *P* = 0.011), whereas type 3 RCH was associated with lower odds of leakage than type 1 (OR: 0.083; 95% CI: 0.026 to 0.267; *P* < 0.001).

**Conclusions:**

A screening strategy integrating UWF-OCTA and UWF-CFP, instead of 150° single-capture UWF-OCTA alone, is reliable for non-invasive detection of RCHs in patients with VHL. OCTA-derived features, particularly the morphological subtype, may replace FA in assessing RCH activity and guiding the management of ocular VHL.

**Supplementary Information:**

The online version contains supplementary material available at 10.1186/s40662-026-00489-x.

## Background

Von Hippel–Lindau disease (VHL) is a rare autosomal dominant hereditary neoplastic syndrome that results from mutations in the *VHL* gene [1]. It is characterized by the development of benign and malignant tumours with multisystem involvement, including retinal capillary haemangiomas (RCHs). Thorough evaluation of the RCH burden is vital in patients diagnosed with VHL, especially in those with poor compliance to follow-up or delayed reporting of visual symptoms (such as children). However, in eye centers that are inexperienced in managing VHL-RCHs, appropriate evaluation is challenging for at least two reasons. First, according to previous studies, 29%‒42% of RCHs are located in the far peripheral retina, anterior to the equator [2, 3]. Second, tiny peripheral RCHs (diameter ≤ 0.5 mm) may present as red or greyish pinpoints, similar to a microaneurysm or punctate intraretinal haemorrhage, and are easily missed [4]. Although dilated fundoscopy is the most accessible method for detecting RCHs, it relies on the examiner’s experience and carries a high risk of missing RCHs [5, 6]. Therefore, multimodal imaging techniques with an ultra-widefield (UWF) of view that provide high-resolution images of the vasculature could be useful for assessing ocular VHL.

UWF fluorescein angiography (UWF-FA) is currently the most sensitive tool for detecting RCHs in patients with VHL [5]. However, in view of the contraindications due to allergy to fluorescein or renal insufficiency following nephrectomy, and the lifelong risk of developing new RCHs, this invasive examination cannot be performed in all patients with VHL or at every follow-up visit. UWF colour fundus photography (UWF-CFP) is a dye-free imaging method that allows clinicians to record the pan-retina with gaze steering non-invasively. However, pseudocolour images and decreased peripheral resolution complicate the definitive diagnosis of suspected lesions, for example, in the context of RCH [7].

Optical coherence tomography angiography (OCTA) is another non-invasive imaging modality that outperforms CFP by providing a highly detailed delineation of the retinal microvasculature and avoids the risks of FA (e.g. anaphylaxis). OCTA is beneficial for morphological classification and navigation of laser photocoagulation in early-stage RCHs [8, 9]. However, conventional OCTA captures a relatively limited field of view compared with UWF-CFP and UWF-FA, which greatly offsets its advantages faced with vascular diseases that predominantly affect the peripheral retina. Recently, two non-exclusive approaches have led to the advent of widefield OCTA: montage and expansion of a single-shot field of view [10]. The widely adopted montage approach generates a total view of approximately 80° by combining five 12 × 12 mm scans with five visual fixations [6, 11]. However, the loss of fixation due to the long acquisition time and the difficulty in ensuring that the entire region is kept in focus throughout the scan duration due to eyeball curvature frequently result in artefacts, especially in the periphery, during montage creation [12]. Moreover, the RCH detection rate across the whole retina is much lower using widefield OCTA than using FA [6]. Thus, OCTA has limited clinical utility for ocular VHL screening. Notably, the commercially available OCTA system (Intalight Dream; Intalight, Luoyang, China) not only characterizes small vascular anomalies with high resolution [13], but also captures the widest field of view (150° eye angle) without requiring a montage when using the most contemporary UWF device [14] and holds promise for detecting RCHs.

Considering the distribution of RCHs in patients with VHL and the complementary advantages of different multimodal imaging techniques, we used a screening method that integrates UWF-CFP and UWF-OCTA for global screening and regional verification, respectively. In this study, we compared the detection rates obtained using single-capture UWF-OCTA and UWF-OCTA plus UWF-CFP with those obtained using UWF-FA and investigated whether UWF-OCTA plus UWF-CFP is a viable non-invasive alternative to UWF-FA for detecting RCHs. Moreover, we delineated the multimodal imaging features of RCHs across the entire retina and investigated the potential associations between non-invasive RCH features and fluorescent leakage.

## Methods

### Study participants

This study included consecutive patients who were clinically and/or genetically diagnosed with VHL at the tertiary referral center Eye and ENT Hospital of Fudan University between 2024 and 2025. The study was conducted in accordance with the tenets of the Declaration of Helsinki and approved by the Institutional Review Board of the Eye and ENT Hospital of Fudan University, Shanghai, China (2020119-1). Demographic and clinical data were retrieved from medical records. The patients underwent comprehensive ophthalmic examinations including visual acuity (VA), slit-lamp biomicroscopy, and UWF imaging. Patients were excluded if they had contraindications or did not require UWF-FA. In our centre, UWF-FA is not recommended to patients with VHL but without suspected RCH on non-invasive examination, because UWF-FA is invasive and requires an appointment. Both eyes of each patient were evaluated. However, we excluded eyes with severe media opacities precluding clear visualization of the retina and eyes with severe motion artefacts in the UWF-OCTA scan due to loss of fixation.

### Image acquisition

In the first step, after pupil dilation with tropicamide 0.5% eye drops, one examiner (XZ) performed single-capture UWF-OCTA (VG200; Intalight) spanning a fovea-centered region measuring 29 × 24 mm, equivalent to a 150° eye angle [14]. If necessary, the examiner gently lifted the upper eyelid with informed consent before imaging. This device uses a swept-source laser with an A-scan rate of 400 kHz, a central wavelength of 1050 nm, an axial resolution of 3.8 μm, and a lateral resolution of 10 μm. OCTA images were acquired using the Intalight transdimensional reconstructed ultrasensitive enhanced angio algorithm. Eye motion artefacts were minimized by using the real-time eye-tracking function of an integrated confocal high-brightness scanning laser ophthalmoscope. A 29 × 24 mm image was obtained from 1280 horizontal B-scans, where each B-scan consisted of 1034 A-scans. The scanning process was repeated twice. When single-capture en-face OCTA imaging of the eyes revealed suspicious RCH, an additional 6 × 6 mm scan of the target region was performed. It comprised 512 horizontal B-scans and 512 A-scans per B-scan.

In the second step, UWF-CFP (California SA; Optos, Dunfermline, Scotland) images were captured orthogonally and with the gaze directed up, down, left, and right. Additional 6 × 6 mm scans were also obtained if RCH was suspected on the UWF-CFP images. An RCH was verified if the internal blood flow signal was detected by both en-face OCTA and OCTA B-scans.

Finally, UWF-FA (California SA; Optos) was recommended to patients if non-invasive imaging detected at least one RCH in either eye and was completed within 1 week. Therefore, the examiner (XZ) was blinded to the UWF-FA findings.

### Image analysis, establishment of a reference standard, and characterization of RCHs

The RCHs were evaluated by one observer (XZ) using single-capture UWF-OCTA and UWF-OCTA plus UWF-CFP and by two observers (YZ and FG) using UWF-FA independently. The number of RCHs detected by UWF-OCTA plus UWF-CFP was calculated as the sum of those detected by single-capture UWF-OCTA and the additional RCHs suspected on UWF-CFP and further verified by regional UWF-OCTA scans. Conglomerate lesions were counted individually if they did not touch each other. Two senior retinal specialists (WL and GX) experienced in managing patients with ocular VHL subsequently adjudicated on any case of discrepancy and established a reference standard for eyes affected by RCH and the total number of RCHs using the original multimodal images combined with the observers’ marks and additional clinical information if available.

The RCHs were characterised as follows: In terms of location, juxtapapillary RCHs were defined as those located within one disc diameter of the disc margin, and regarding the anteroposterior location, we used zonal criteria previously developed for cytomegalovirus retinopathy by Gary et al. [15]. According to these criteria, zone 1 refers to the retina within the major temporal vascular arcades, including the juxtapapillary area. Zone 3 is the retinal area anterior to the vortex vein ampulla and up to the ora serrata. Zone 2 refers to the area between zones 1 and 3. Although the primary goal of this study was to detect tiny RCHs (≤ 0.5 mm), we noted that some larger RCHs were also difficult to detect, especially those located in zone 3. Thus, the RCHs were also classified by size: ≤ 0.5 mm or > 0.5 mm.

**Fig. 1:**
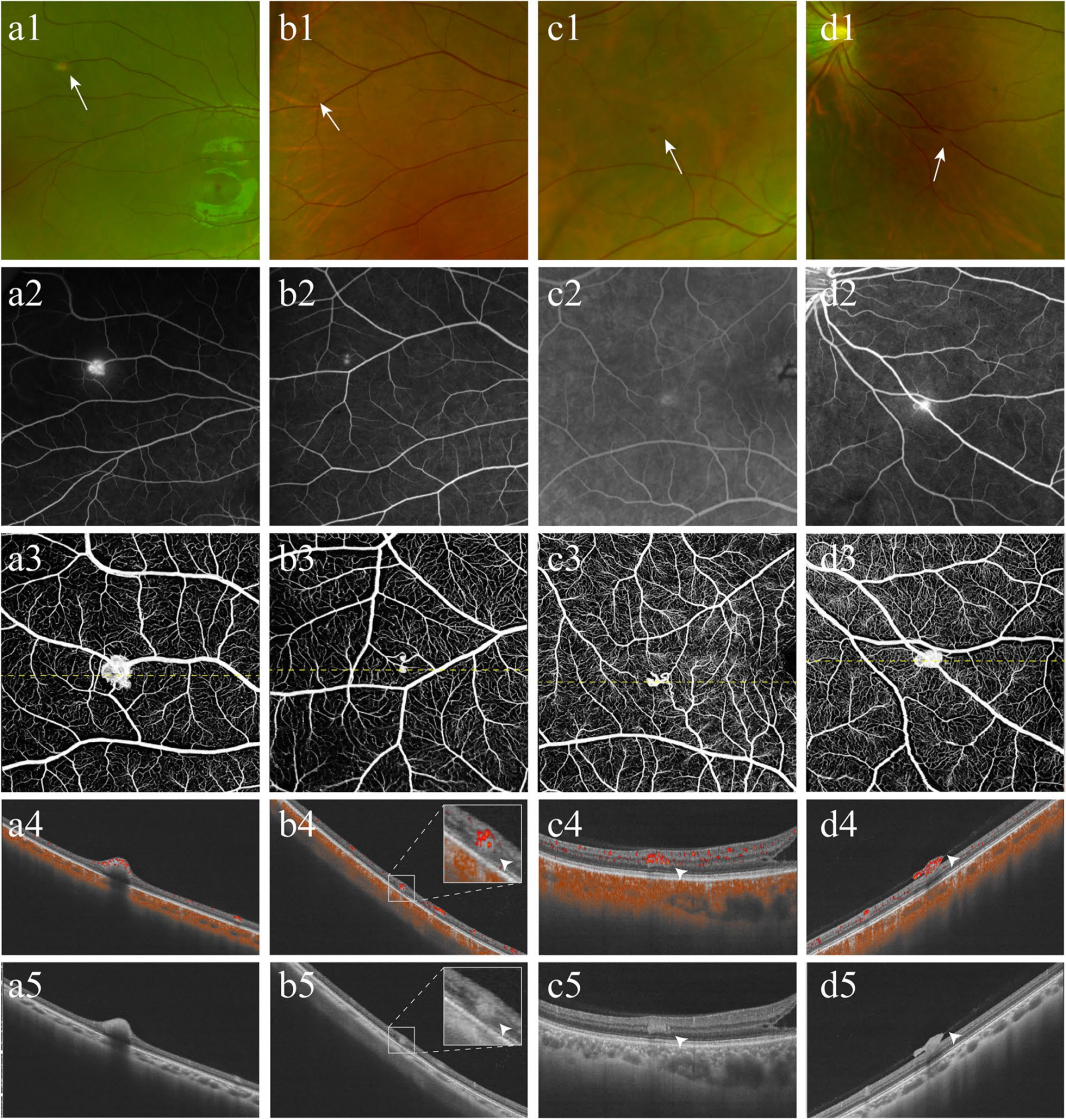
Representative OCTA-classified subtypes of RCHs. Subtypes of RCHs with multimodal imaging techniques, including UWF-CFP (**a1**‒**d1**), UWF-FA (**a2**‒**d2**), and en-face OCTA (**a3**‒**d3**), along with OCTA B-scan (**a4**‒**d4**) and OCT B-scan (**a5**‒**d5**), are shown. Type 1 RCH presents as an orange nodular lesion (**a1**, arrow), showing hyperfluorescence with late leakage on UWF-FA (**a2**) and dense vascular cluster-like changes on en-face OCTA (**a3**). It is characterised by protrusion of the superficial retina into the vitreous cavity (**a4** and **a5**). Type 2 appears faint grey–white (**b1**, arrow) and shows hyperfluorescence without leakage on UWF-FA (**b2**) and a globular blood flow signal on en-face OCTA (**b3**). It compresses the adjacent ONL and disrupts the EZ underneath (**b4** and **b5**, arrowheads; magnified view is shown in the inset). Type 3 RCH manifests as a red dot-like lesion (**c1**, arrow), whose fluorescence is difficult to differentiate from that of the background (**c2**). En-face OCTA delineates the blood-flow-rich tangled contour (**c3**). However, it is flat with a relatively intact ONL and EZ (**c4** and **c5**, arrowheads). Type 4 RCH appears faint grey–white overlying the vessel (**d1**, arrow), with hyperfluorescent leakage (**d2**) and a detectable blood flow signal (**d3**). OCTA/OCT B-scans demonstrate that it breaches the inner limiting membrane and grows along the posterior vitreous cortex (**d4** and **d5**, arrowheads). CFP, colour fundus photography; EZ, ellipsoid zone; FA, fluorescein angiography; OCTA, optical coherence tomography angiography; ONL, outer nuclear layer; RCH, retinal capillary haemangioma; UWF, ultra-widefield

Building upon the classic categorization [16] and our previous observation of juxtapapillary RCHs [17], the OCTA B-scan-based RCH classification incorporates the following factors: breaching of the inner limiting membrane (ILM), mass effect (nodular compression), and secondary changes in the superficial and outer retinas, suggesting the main direction of growth. In this classification system, RCHs that protrude into the vitreous cavity without breaching the ILM are designated type 1. RCHs without marked superficial retinal elevation but with compression of the outer retina are designated as type 2. In this context, compression of the outer retina is demonstrated by disruption of the outer nuclear layer and/or ellipsoid zone. In contrast to types 1 and 2, which share a nodular appearance with a presumed mass effect, type 3 RCHs are flat and located in any layer of the retina without obvious signs of compression. Unlike types 1‒3, which are all intraretinal, type 4 RCHs breach the ILM, manifest as preretinal neovascularization-like tufts, and grow in the vitreous cavity. Representative images of the four subtypes are shown in Fig. 1.

### Statistical analyses

Statistical analyses were performed using SPSS 20.0 (IBM Corp). Snellen VA was converted to the logarithm of the minimum angle of resolution (logMAR) units for statistical analyses. The Shapiro–Wilk test was used to evaluate normality. Continuous data are presented as mean ± standard deviation (SD) or as median with interquartile range (IQR) and range. Categorical data are presented as numbers and percentages. Interobserver reproducibility was assessed using Cohen’s κ coefficient for eyes with RCH and intraclass correlation coefficient (ICC) for RCH number, which was based on a single-measures, absolute-agreement, two-way random-effects model. ICC and κ were interpreted as previously classified: 0.0–0.20, slight agreement; 0.21–0.40, fair agreement; 0.41–0.60, moderate agreement; 0.61–0.80, substantial agreement; 0.81–1.00, almost-perfect agreement [18]. For eye-level analysis, we used the generalised estimating equation model to account for repeated measurements in both eyes. For RCH-level analysis, we used a generalized linear mixed model (GLMM) to account for the inclusion of both eyes per individual and the correlations between multiple RCHs per eye. We also used the GLMM to identify potential non-invasive imaging features of individual RCHs that could predict FA leakage. Factors with an overall *P* value of < 0.1 in the univariable analyses were included in the multivariate model. The analyses were adjusted for multiple comparisons using the Bonferroni method. *P* values of < 0.05 were considered statistically significant.

## Results

### Demographic and clinical characteristics of the patients

Twenty-eight consecutive patients with VHL underwent UWF-OCTA and UWF-CFP to detect RCHs. Of these, UWF-FA was not performed in seven patients for the following reasons: five patients (17.9%) had no evidence of RCH in either eye, one patient (3.6%) with RCHs had a history of nephrectomy-related renal insufficiency, and one patient (3.6%) with RCHs declined invasive examinations.

Therefore, the study comprised 21 patients (mean ± SD age: 34.6 ± 11.3 years; 13 [61.9%] men and 8 [38.1%] women) who underwent UWF-OCTA, UWF-CFP, and UWF-FA. Ten patients (47.6%) without ocular symptoms were referred to our center for multidisciplinary surveillance of VHL, and two patients (9.5%) with blindness in one eye due to severe complications of uncontrolled RCHs attended our center for regular examination of their asymptomatic eyes. The other nine patients were symptomatic, of whom six (28.6%) presented with blurred vision, two (9.5%) had floaters and flashes, and one (4.8%) developed visual field defects.

Of the 42 evaluated eyes, data were unavailable for three eyes because imaging could not be performed in two eyes due to severe neovascular glaucoma, and the UWF-OCTA scan was ungradable in one eye because of severe tractional retinal detachment and motion artefacts. Therefore, 39 eyes with UWF-CFP, UWF-OCTA, and UWF-FA images of sufficient quality were included in the analysis. The median VA was 0.05 (IQR: 0‒0.1; range: 0‒1.3) logMAR.

### Overall multimodal imaging features of RCHs

**Fig. 2:**
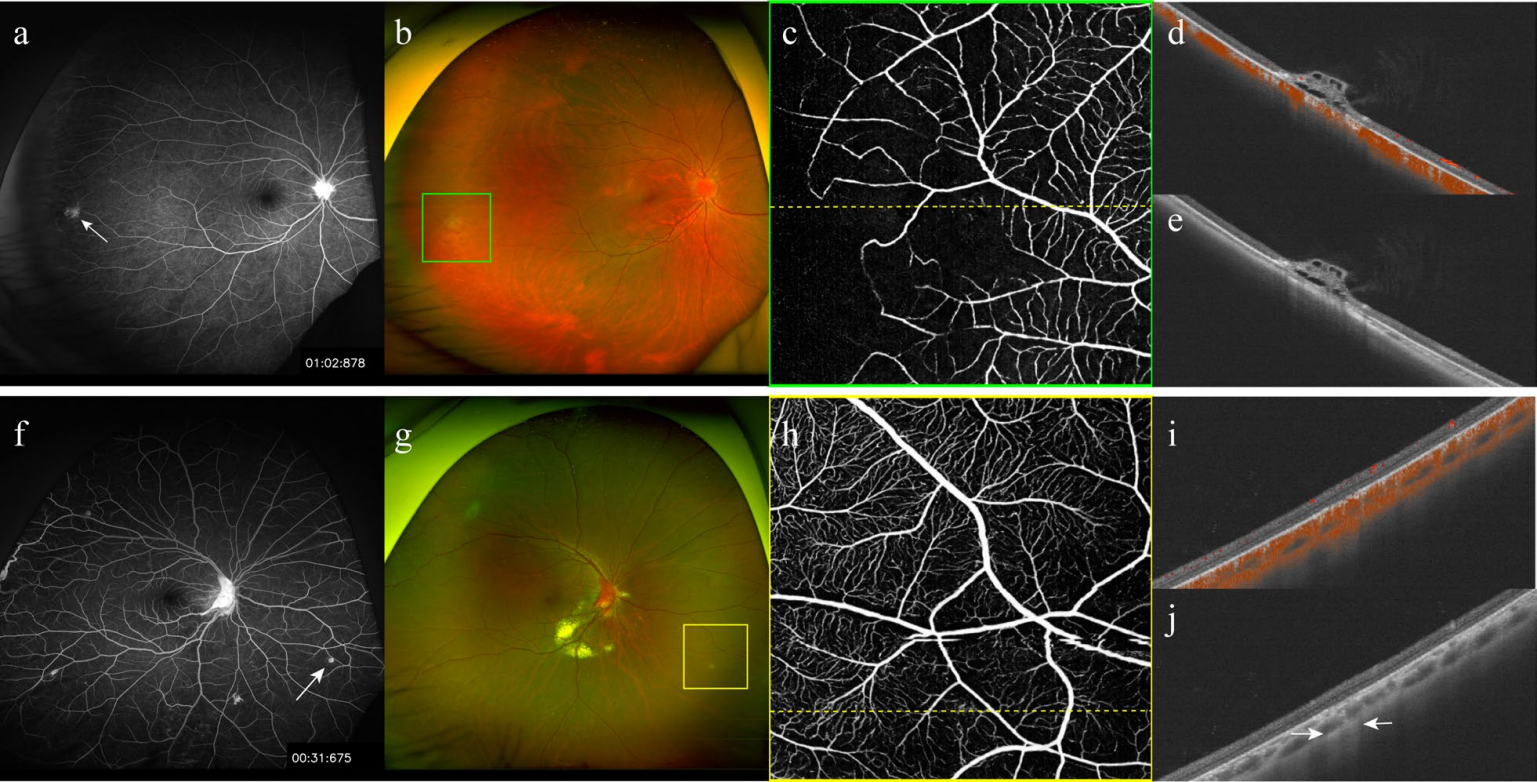
Examples of RCH-mimicking lesions in patients with VHL. **a**‒**e** Case 1. **a** UWF-FA reveals a hyperfluorescent lesion on the temporal side (arrow), which presents as a slightly bulging grey–white lesion on UWF-CFP (**b**). **c**, **d** Regional en-face OCTA (**c**) or OCTA B-scan (**d**) along the yellow dashed line in (**c**) does not reveal internal vascularity. **e** Retinal elevation with hypo-reflective cavities on the OCT B-scan suggests a cystic retinal tuft. **f**‒**j** Case 2. **f** A hyperfluorescent lesion (arrow) adjacent to a vessel in the inferonasal quadrant on UWF-FA. The lesion is pale on UWF-CFP (**g**) and does not show any evidence of blood flow signal on regional en-face OCTA (**h**) or OCTA B-scan (**i**). A transmission effect is observed on the OCT B-scan (arrows), suggesting retinal pigment epithelium anomaly. CFP, colour fundus photography; FA, fluorescein angiography; OCTA, optical coherence tomography angiography; RCH, retinal capillary haemangioma; UWF, ultra-widefield; VHL, von Hippel–Lindau disease

All the presumed RCH lesions were carefully reviewed by senior specialists. Six lesions were judged RCH-mimicking and were excluded from further analysis. The spectrum of RCH-mimicking lesions ranged from retinal pigment epithelium anomalies to cystic retinal tufts; examples of these are shown in Fig. 2. Subsequently, the reference standard revealed bilateral involvement in 81% of patients (17/21) and 151 RCHs in 89.7% of eyes (35/39), with a median of two (IQR: 1‒3; range: 0‒29) per eye.

The overall multimodal imaging features of confirmed RCHs are summarised in Table 1. Most of the RCHs were in zones 2 (48.3%) and 3 (43.0%), ≤ 0.5 mm in size (69.5%), and red–orange in colour (74.2%). Most RCHs were hyperfluorescent on UWF-FA, with leakage (67.5%) or without leakage (29.8%). Based on OCTA B-scans, RCHs can be classified into four subtypes. Most were nodular RCHs, corresponding to types 1 (48.8%) and 2 (18.1%). Unlike types 1 and 2 RCHs, type 3 RCHs (28.3%) were characterised by creeping growth and a flat appearance. However, type 3 RCHs can be easily identified using OCTA. Type 4 RCHs, which present as preretinal neovascularization-like tufts, are rare and comprise only 4.7% of the detected RCHs. UWF-FA did not reveal any non-perfused area across the entire retina in eyes with type 4 RCHs.

**Table 1:** General multimodal imaging characteristics of RCHs (n = 151)

Imaging characteristics	Results
Circumferential location	
Superotemporal	49 (32.5)
Inferotemporal	35 (23.2)
Superonasal	27 (17.9)
Inferonasal	30 (19.9)
Juxtapapillary	10 (6.6)
Anteroposterior location	
Zone 3	65 (43.0)
Zone 2	73 (48.3)
Zone 1	13 (8.6)
Largest basal diameter	
≤ 0.5 mm	105 (69.5)
> 0.5 mm	46 (30.5)
FA feature	
Isofluorescence	4 (2.6)
Hyperfluorescence without leakage	45 (29.8)
Hyperfluorescence with leakage	102 (67.5)
Colour	
Red–orange	112 (74.2)
Grey–white	24 (15.9)
Unremarkable	15 (9.9)
OCTA B-scan morphological type (n = 127^†^)	
Type 1	62 (48.8)
Type 2	23 (18.1)
Type 3	36 (28.3)
Type 4	6 (4.7)

*CFP* = colour fundus photography; *FA* = fluorescein angiography; *OCTA* = optical coherence tomography angiography; *RCH* = retinal capillary haemangioma; *UWF* = ultra-widefield

Data are numbers with percentage in parentheses

^†^Except for twenty RCHs missed using UWF-OCTA plus UWF-CFP, another four RCHs were identified only using UWF-CFP without OCTA

### Detection of RCHs on single-capture UWF-OCTA and UWF-OCTA plus UWF-CFP versus UWF-FA

**Fig. 3:**
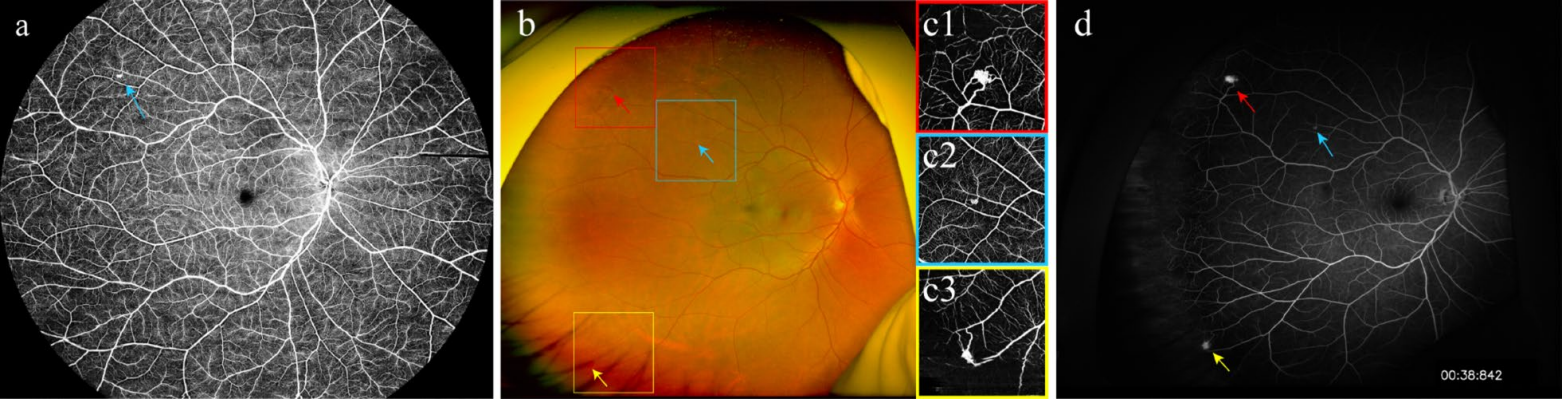
Comparison of RCH detection on single-capture UWF-OCTA or UWF-OCTA plus UWF-CFP versus UWF-FA. **a** Single-capture 150° UWF-OCTA detects a tiny RCH in the superotemporal quadrant (blue arrow). **b**, **c** UWF-CFP with temporal gaze steering shows the single-capture UWF-OCTA**-**detected lesion (**b**, blue arrow) and two additional suspected RCHs (**b**, red and yellow arrows), all of which are verified as RCHs by regional 6 × 6 mm en-face OCTA (**c1**‒**c3**). **d** They are hyperfluorescent without late leakage on UWF-FA (blue, red, and yellow arrows). CFP, colour fundus photography; FA, fluorescein angiography; OCTA, optical coherence tomography angiography; RCH, retinal capillary haemangioma; UWF, ultra-widefield

We compared the RCH detection performances of single-capture UWF-OCTA, UWF-OCTA plus UWF-CFP, and UWF-FA (Table 2), and the representative images are presented in Fig. 3. Agreement between two observers on UWF-FA was substantial for detecting eyes with RCH (κ = 0.721) and almost perfect for the number of RCHs per eye (ICC = 0.867). At the eye level, at least one RCH was detected in 89.7% (95% CI: 76.4%‒95.9%) of eyes using UWF-FA, 61.5% (95% CI: 45.9%‒75.1%) using single-capture UWF-OCTA, and 87.2% (95% CI: 73.3%‒94.4%) using UWF-OCTA plus UWF-CFP. The median number of RCHs per eye was 2 (IQR: 1–3; range: 0‒27) detected by UWF-FA, 1 (IQR: 0–2; range: 0‒15) by single-capture UWF-OCTA, and 2 (IQR: 1–3; range: 0‒24) by UWF-OCTA plus UWF-CFP. With UWF-FA set as the reference, the detection rate and number of RCHs per eye were significantly lower for single-capture UWF-OCTA (*P* < 0.001 and *P* = 0.003, respectively), but not for UWF-OCTA plus UWF-CFP (*P* = 0.319 and 0.252, respectively). At the RCH level, among the 151 RCHs per reference standard, 141 were recognised by UWF-FA, 78 by single-capture UWF-OCTA, and 131 by UWF-OCTA plus UWF-CFP. The detection rate of RCHs using single-capture UWF-OCTA was significantly lower than that using UWF-FA (51.7% [95% CI: 43.7%–59.5%] vs. 93.4% [95% CI: 88.2%–96.4%]; *P* < 0.001). Although the detection rate using UWF-OCTA plus UWF-CFP was lower than that using UWF-FA, the difference was not statistically significant (86.8% [95% CI: 80.4%–91.3%] vs. 93.4% [95% CI: 88.2%–96.4%]; *P* = 0.057).

**Table 2:** Comparison of detecting RCHs by single-capture UWF-OCTA or UWF-OCTA plus UWF-CFP versus UWF-FA

Parameter	Screening method				
	UWF-FA	Single-capture UWF-OCTA	UWF-OCTA plus UWF-CFP	*P*^†^	*P*^§^
Eye level (n = 39)					
Diagnosis of at least one RCH	35 (89.7) [76.4‒95.9]	24 (61.5) [45.9‒75.1]	34 (87.2) [73.3‒94.4]	** < 0.001**	0.319
Number of RCHs per eye^*^	2 (1‒3, 0‒27)	1 (0‒2, 0‒15)	2 (1‒3, 0‒24)	**0.003**	0.252
RCH Level (n = 151)					
Detected RCHs	141 (93.4) [88.2‒96.4]	78 (51.7) [43.7‒59.5]	131 (86.8) [80.4‒91.3]	** < 0.001**	0.057

*CFP* = colour fundus photography; *FA* = fluorescein angiography; *OCTA* = optical coherence tomography angiography; *RCH* = retinal capillary haemangioma; *UWF* = ultra-widefield

Unless otherwise specified, data are numbers with percentage in parentheses and 95% confidence interval in brackets

*P* values in bold are statistically significant

^†^Single-capture UWF-OCTA versus UWF-FA

^§^UWF-OCTA plus UWF-CFP versus UWF-FA

^*^Data are medians, with interquartile range and range in parentheses

We separately analysed 20 RCHs that were missed on UWF-OCTA plus UWF-CFP. The features of these RCHs are summarised in Additional File 1. Generally, the missed RCHs were more commonly located in zone 3 (85%), ≤ 0.5 mm in size (85%), and red–orange (65%) or unremarkable (25%) in colour on UWF-CFP. In UWF-FA, 55% of the RCHs were hyperfluorescent without leakage, whereas the others were hyperfluorescent with leakage.

### Correlations between non-invasive multimodal features and FA leakage

Finally, we examined the potential non-invasive factors that predicted RCH activity, which was previously reflected in FA-based hyperfluorescence with leakage (Table 3). The multivariate GLMM revealed that RCH size of > 0.5 mm significantly increased the likelihood of fluorescent leakage (OR: 10.987; 95% CI: 1.747‒69.090; *P* = 0.011). Additionally, the OCTA subtype was significantly correlated with fluorescent leakage (*P* = 0.001), and type 3 RCH was associated with a lower risk of fluorescent leakage than type 1 RCH (OR: 0.083; 95% CI: 0.026‒0.267; *P* < 0.001). Other RCH features, including circumferential and anteroposterior locations and colour, were not significantly associated with leakage.

**Table 3:** Univariable and multivariate logistic regression analysis of factors associated with hyperfluorescence with leakage

Factor	Univariable analysis			Multivariate analysis^†^		
	OR	95% CI	*P*	OR	95% CI	*P*
Circumferential location	–	–	0.667	–	–	–
Superotemporal	Ref.	–	–	–	–	–
Inferotemporal	1.058	0.408‒2.745	0.907	–	–	–
Superonasal	0.824	0.301‒2.259	0.705	–	–	–
Inferonasal	0.837	0.315‒2.225	0.720	–	–	–
Juxtapapillary	4.364	0.491‒38.777	0.185	–	–	–
Anteroposterior location	–	–	0.126	–	–	–
Zone 3	Ref.	–	–	–	–	–
Zone 2	2.020	0.971‒4.199	0.060	–	–	–
Zone 1	2.369	0.581‒9.651	0.227	–	–	–
Largest basal diameter	–	–	-	–	–	–
≤ 0.5 mm	Ref.	–	–	Ref.	–	–
> 0.5 mm	29.557	4.956‒176.277	** < 0.001**	10.987	1.747‒69.090	**0.011**
Colour	–	–	**0.026**	–	–	0.289
Red–orange	Ref.	–	–	Ref.	–	–
Grey–white	0.971	0.361‒2.613	0.954	0.576	0.145‒2.290	0.431
Unremarkable	0.200	0.062‒0.645	**0.007**	0.198	0.021‒1.866	0.156
OCTA B-scan morphologic subtype	–	–	** < 0.001**	–	–	**0.001**
Type 1	Ref.	–	–	Ref.	–	–
Type 2	0.311	0.088‒1.096	0.069	0.396	0.108‒1.450	0.160
Type 3	0.049	0.016‒0.147	** < 0.001**	0.083	0.026‒0.267	** < 0.001**
Type 4	3.717	0.027‒503.307	0.597	2.626	0.033‒208.299	0.663

*CI* = confidence interval; *OCTA* = optical coherence tomography angiography; *OR* = odds ratio

*P* values in bold are statistically significant

^†^After adjustment for tumour diameter, colour, and OCTA subtype

## Discussion

We described the comprehensive retinal imaging features of patients with VHL using multiple UWF techniques, investigated their ability to detect RCHs, and determined the correlations between multimodal imaging characteristics. The reference standard revealed a median of 2 RCHs per eye, which was higher than the mean of 0.74 RCHs in a previous report [6]. Moreover, bilateral involvement was found in more than 80% of the enrolled patients, exceeding the rate of 57.9% among patients with VHL-RCH in at least one eye reported in a prior study [19]. The combination of multiple UWF imaging techniques explains this observed discrepancy. This alerted us to the possibility of missing RCHs, even in patients with VHL without ocular symptoms. Once RCH is detected in one eye, careful examination of the contralateral eye is necessary because bilateral involvement is much more common than expected. A detailed dilated fundoscopic examination by a trained specialist is required when UWF imaging techniques are unavailable.

Every imaging modality and every scanning model has appropriate applications. For example, single-capture widefield OCTA shows high sensitivity for detecting retinal neovascularization and diagnosing proliferative diabetic retinopathy [20, 21]. However, according to our results, the capacity of 150° single-capture UWF-OCTA for detecting RCHs remains insufficient. This situation arises from the fact that 43% of the RCHs are located in zone 3, which is consistent with previous reports [2]. Regarding UWF-CFP, Li et al. reported that its sensitivity for detecting peripheral lesions reached 86.5% in concert with mydriatic eye steering [22]. Therefore, we adopted UWF-CFP with eye steering as a global screening approach for patients with VHL. However, peripheral lesions, including holes and tears, are easy to recognize and diagnose, whereas tiny RCHs may only be suspected rather than verified using UWF-CFP. Therefore, a screening strategy that integrates multiple imaging methods and optimises the advantages of each is required.

In real-world practice, applying UWF-OCTA to suspicious lesions identified using UWF-CFP improves the reliability of RCH detection. We found that UWF-OCTA plus UWF-CFP yielded a slightly lower RCH detection rate than UWF-FA, which is the most sensitive tool for ocular VHL [5], although the difference was not statistically significant. However, this screening method avoids long acquisition time, low patient compliance, peripheral artefacts with UWF-OCTA montaging, and contraindications for UWF-FA. In addition, some RCHs display only mild hyperfluorescence or isofluorescence on UWF-FA, and are easily missed [6]. Furthermore, some RCH-mimicking lesions show false-positive hyperfluorescence on UWF-FA, making treatment decisions more challenging [23]. Nevertheless, OCTA plays a pivotal role in determining whether the equivocal lesions are RCHs. Such lesions can be successfully identified or excluded based on OCTA blood flow signals, which is a cardinal characteristic of RCH. For example, a cystic retinal tuft can be diagnosed in the context of VHL based on the absence of blood flow signal on UWF-OCTA (Fig. 2a‒e). However, 20 RCHs (13.2%) could not be detected using this method. The reasons for missing RCHs include the unremarkable appearance of small RCHs in pseudocolour images and the failure to capture those in the far periphery, especially with insufficient eye steering on UWF-CFP. Given that the suspected RCHs on UWF-CFP were successfully verified or excluded by UWF-OCTA, OCTA artefacts made little contribution to the missing rate of RCHs, although OCTA artefacts including eyelash artefacts cannot be completely avoided [24]. Fortunately, all missed RCHs were tiny (≤ 0.5 mm) and therefore unlikely to grow to a difficult-to-treat size before the next regular follow-up. Overall, our data suggest that although UWF-OCTA plus UWF-CFP is imperfect, it is a reliable, time-saving, and non-invasive alternative to UWF-FA for detecting RCHs.

Traditionally, RCH classification has been based on CFP and FA. Juxtapapillary RCHs can be subgrouped into endophytic, sessile, and exophytic types [16]. An outstanding advantage of OCTA is that it can provide a more precise classification, owing to the capacity of the B-scan for cross-sectional observation. Ayako et al. described a flat RCH type with presumed indolent behaviour within the macular and mid-peripheral regions [25]. Similar to the flat type proposed by Ayako et al., type 3 RCHs were identified more prominently using UWF-OCTA. However, given the cross-sectional nature of the study, we are unsure whether type 3 RCH represents an early stage of type 1 or type 2 and whether it will stay “silent”, which warrants long-term follow-up. Importantly, type 4 RCHs are likely to be mistaken for ischaemia-driven retinal neovascularization. An example of neovascularization over a large RCH was reported and postulated to arise from vascular steal-related nonperfusion [26]. However, UWF-FA did not reveal peripheral nonperfusion in any eye with type 4 RCH, suggesting that type 4 RCH is an independent subtype. In general, this OCTA-based RCH classification has advanced our understanding of RCHs and laid the foundation for improving the management of ocular VHL.

UWF-FA is not only useful for detecting lesions but also for evaluating disease activity [27]. We considered that hyperfluorescence with leakage represents greater activity than that without leakage. Leakage implies a higher likelihood of tumour growth with secondary changes such as retinal oedema, which justifies earlier intervention. According to multivariate analysis, tumour size and OCTA subtype were significantly associated with leakage. Type 3 RCHs were less likely to be accompanied by FA leakage than type 1 RCHs, which were the most common. The lack of leakage in type 3 RCHs supports their indolence, at least at the time of imaging. According to the guidelines for ocular VHL, more frequent follow-up is recommended if observation is chosen instead of early treatment, even for small peripheral RCHs (≤ 0.5 mm) [28]. Our results suggest that non-invasive features such as the OCTA subtype, which is predictive of RCH activity, can be incorporated into the decision-making process. Future studies may explore OCTA-guided management of small RCHs without FA-derived information.

This study had some limitations. First, UWF-FA, UWF-CFP, and especially UWF-OCTA images were available for only a small number of patients owing to the rarity of VHL and the recent advent of UWF-OCTA. Some of the calculated post hoc powers were smaller than 0.8, suggesting a potential risk of type II statistical error. In other words, the limited sample size may have resulted in insufficient recognition of potential differences. Larger multicentre studies are warranted to validate the results of the present study. Second, as a retrospective cross-sectional study, some patients without evidence of RCHs on UWF-CFP and UWF-OCTA did not undergo UWF-FA, which may have introduced selection bias and overestimated the detection rate. Additionally, eyes with poor fixation and severe eye motion artefacts on OCTA were excluded. However, excluding such eyes may also introduce selection bias, which may limit the generalisability of our results. Thus, the conclusions should be limited to patients with preserved fixation and cannot be extended to those with very poor vision or nystagmus.

## Conclusions

In summary, UWF-OCTA plus UWF-CFP achieved an overall detection rate of 86.8% for RCHs and may serve as a feasible non-invasive screening alternative for ocular VHL, especially when patients have contraindications to UWF-FA. Moreover, OCTA B-scan-based RCH classification can help predict fluorescein-dependent RCH activity, which may be used for ocular VHL surveillance. In the future, artificial intelligence models could be developed using UWF multimodal images to detect RCHs automatically, predict their activity, and customize follow-up intervals and treatment recommendations.

## Supplementary Information

Additional file 1.

## Data Availability

The datasets used and/or analysed in the current study are available from the corresponding author upon reasonable request.

## Abbreviations

CFP: Colour fundus photography
CI: Confidence interval
FA: Fluorescein angiography
GLMM: Generalized linear mixed model
ICC: Intraclass correlation coefficient
ILM: Inner limiting membrane
IQR: Interquartile range
OCTA: Optical coherence tomography angiography
OR: Odds ratio
RCH: Retinal capillary haemangioma
SD: Standard deviation
UWF: Ultra-widefield
VA: Visual acuity
VHL: Von Hippel–Lindau disease
