# Supplementary material for: Ultra-widefield optical coherence tomography angiography plus colour fundus photography in von Hippel‒Lindau disease: detection, classification, and correlations with findings on fluorescein angiography
Source: Eye Vis (Lond). 2026 May 14;13:19. doi: 10.1186/s40662-026-00489-x (PMC13173854; doi:10.1186/s40662-026-00489-x)
Supplement: Supplementary file 1 — Additional file 1. [file 40662_2026_489_MOESM1_ESM.docx]

**Additional File 1. Features of RCHs Missed on UWF-OCTA plus UWF-CFP (*n* = 20)**

| Feature | Results |
| --- | --- |
| Circumferential location |  |
| Superotemporal | 8 (40) |
| Inferotemporal | 1 (5) |
| Superonasal | 6 (30) |
| Inferonasal | 5 (25) |
| Juxtapapillary | 0 (0) |
| Anteroposterior location |  |
| Zone 1 | 0 (0) |
| Zone 2 | 3 (15) |
| Zone 3 | 17 (85) |
| Largest basal diameter |  |
| ≤ 0.5 mm | 17 (85) |
| > 0.5 mm | 3 (15) |
| FA feature |  |
| Isofluorescence | 0 (0) |
| Hyperfluorescence without leakage | 11 (55) |
| Hyperfluorescence with leakage | 9 (45) |
| Color |  |
| Red-orange | 13 (65) |
| Grey-white | 2 (10) |
| Unremarkable | 5 (25) |

Abbr: CFP, colour fundus photography; FA, fluorescein angiography; OCTA, optical coherence tomography angiography; RCH, retinal capillary haemangioma; UWF, ultra-widefield.

Data are numbers with percentage in parentheses.
